# Towards a sustainable phosphorus network in Africa

**DOI:** 10.1016/j.soilad.2025.100067

**Published:** 2025-12

**Authors:** M.G. Manzeke-Kangara, M.S.A. Blackwell, W.J. Brownlie, E. Zaja, K.A. Frimpong, A.A. Asante, D. Cordell, J.J. Elser, E.K. Gbekor, K. Harris-Gilliam, D. Touhami, J.X. Kugbe, I.H. Lewis, V. Logah, M. Miyittah, E.K. Nartey, M. Owusu, Z. Oyetunde-Usman, C.K. Tay, F.M. Tetteh, P.M. Haygarth, B.M. Spears

**Affiliations:** a Rothamsted Research, West Common, Harpenden AL5 2JQ, United Kingdom; b UK Centre for Ecology and Hydrology, Edinburgh EH26 0QB, United Kingdom; c African Plant Nutrition Institute (APNI), Morocco; d UK Centre for Ecology and Hydrology, West Africa Office, Accra, Ghana; e Institute for Sustainable Futures, University Technology Sydney, Sydney, Australia; f School of Sustainability, Arizona State University, Tempe, AZ, United States; g Flathead Lake Biological Station, University of Montana, Polson, MT, United States; h International Fertilizer Development Center (IFDC), Accra, Ghana; i The STEPS Centre, North Carolina State University, Raleigh, NC, United States; j AgroBiosciences Program, College of Agriculture and Environmental Sciences, Mohammed VI Polytechnic University (UM6P), Hay Moulay Rachid, Ben Guerir, Morocco; k OCP Africa, Casablanca, Ghana; l Department of Crop and Soil Sciences, Kwame Nkrumah University of Science and Technology, Kumasi, Ghana; m Department of Environmental Science, University of Cape Coast, Cape Coast, Ghana; n Department of Soil Science, School of Agriculture, University of Ghana, Accra, Ghana; o Directorate of Crop Services, Ministry of Food and Agriculture, Accra, Ghana; p Council for Scientific and Industrial Research (CSIR)-Soil Research Institute, Accra, Ghana; q Lancaster Environment Centre, Lancaster University, Lancaster LA1 4YQ, United Kingdom

**Keywords:** Collaborative partnerships, Environmental protection, Environmental sustainability, Food security, Knowledge co-creation and inclusivity, sub–Saharan Africa, Sustainable phosphorus management

## Abstract

Global collaborative action for sustainable management of phosphorus is vital to ensure food production and the protection of water quality. This requires balancing competing phosphorus demands and a growing population through coordinated actions at local, national, regional and international scales. Phosphorus is mainly used in the agricultural sector as an essential nutrient for plant growth and animal feed. It is also used to a lesser extent in the food industry as an additive, as an additive in steel production and most recently in the production of lithium batteries for electric cars. Phosphorus is a finite resource, making its sustainable use a global priority. Yet, losses from the global phosphorus system risk pollution of aquatic ecosystems associated with biodiversity loss and human health risks associated with harmful algal blooms. While phosphorus supplies from reserves are not of immediate concern, there is nonetheless a need to ensure sustainable phosphorus use at the global level. Africa’s use of phosphorus fertilisers is sub-optimal, with a reported decline in phosphorus fertiliser use of about 233 % by the turn of the 21^st^ century, and remains low. The Sustainable Phosphorus Summit (SPS) is the only global conference series supporting discourse on phosphorus sustainability spanning across the academic, agriculture, environmental, wastewater, policy and industry sectors. Since its inception in 2010, the SPS series has been held every 2–4 years on all continents – except Africa. The hosting of the 8^th^ SPS (SPS8) in Accra, Ghana, will present an opportunity to set the agenda for sustainable phosphorus management in Africa, and to place African contexts into the global discussion. Being organised by different teams led by an African Local Organising Committee, SPS8 aims to pave the way towards the establishment of an African Sustainable Phosphorus Network, serving as a platform for collaboration, networking and knowledge co-creation and exchange to ensure sustainable phosphorus use in the region and beyond. Sustainable phosphorus management in Africa is feasible in the medium to long-term, with a focus on ensuring adequate phosphorus fertiliser availability, access and use, while minimising the environmental impacts from losses by matching soil-crop phosphorus needs and enhancing circular phosphorus use systems, and informing ecosystem recovery planning.

## 1 Introduction

### 1.1 Major global phosphorus challenges

The need for global actions to deliver sustainable phosphorus management is well recognised (Brownlie et al., 2021a, Elser and Haygarth, 2021). Phosphorus (together with nitrogen) is one of the key planetary boundaries that has exceeded the safe limits for humanity, particularly as a global water pollutant causing widespread harm (Richardson et al., 2023).

The phosphorus planetary boundary is calculated by estimating the maximum annual anthropogenic phosphorus flow into freshwater systems, that can occur without triggering widespread eutrophication (Steffen et al., 2015, Richardson et al., 2023) and to avoid ecological tipping points (Rockström et al., 2009). While this is a crucial analysis, several assumptions are taken into consideration including globally averaged ecosystem sensitivity, use of simplified models and a lack of accounting for delayed effects from legacy phosphorus in soils and sediments.

At the same time, phosphorus is an essential nutrient required by all farmers to grow crops, yet the world’s main source currently comes from finite reserves (U.S. Geological Survey, 2024). Complexities in access to finite phosphorus resources and the need to produce food sustainably and in an environmentally conscious manner is at the core of the global phosphorus challenge.

In recent years, this has culminated in calls for action including the Helsinki Declaration which saw over 500 scientists signing the Call for International Action on Phosphorus by the end of 2020. This petition calls for government support in addressing phosphorus concerns by coordinating action across five primary sectors (https://www.opfglobal.com/helsinki-declaration), expanded on in detail through the Our Phosphorus Future Project (Brownlie et al., 2022a). Phosphorus is embedded within some global initiatives, including the Kunming-Montreal Global Biodiversity Framework (KM-GBF) (UNEP, 2022). For example, reducing nutrient pollution of water is reflected in Targets 2 and 7 of the KM-GBF, and the sustainable use of fertilisers is also reflected in Targets 7 and 10 of the Framework. In addition, the United Nations Environment Assembly (UNEA) Resolution 5/2 notes concern of the environmental impacts of excessive nitrogen and phosphorus use and recognises the importance of the sustainable use of these nutrients in safeguarding global food security and ending hunger (UNEP/EA.5/Res.2, 2022).

Phosphorus spans a range of global sustainability policy fields, including food and water security, climate change, and biodiversity recovery. Therefore, it is placed at the centre of the Triple Planetary Crisis of biodiversity loss, pollution, and climate change (Miao and Nduneseokwu., 2024). Phosphorus remains essential for meeting the growing food production demands in regions like sub-Saharan Africa, while it is often overused in many developed countries (Cordell et al., 2022).

The supply of phosphorus fertiliser is currently dominated by mined sources and drives a global trade in food and non-food products (Brownlie et al., 2021b). From a supply perspective, reserves are finite. Although the long-term supply of phosphorus from primary natural reserves is not of immediate concern globally, access to phosphate rock on the market is of immediate concern, due to geopolitical disruptions to supply chains and price spikes. This has resulted in some countries legislating for a rebalance in the use of mined phosphorus versus phosphorus recycled from waste streams; informed by phosphorus flow models at the catchment to national scales (Brownlie et al., 2021b). This shift recognises two factors. Firstly, on food system resilience where geopolitics can leave countries exposed to a volatile fertiliser market (Brownlie et al., 2022b). Secondly, on water security, where phosphorus losses through wastewater discharges and agricultural run-off impact freshwater and coastal ecosystems leading to increased human health risks, disruption to drinking water supplies, and a loss of freshwater biodiversity (WWQA Ecosystems, 2023).

Attention on phosphorus management and ecosystem degradation date back to the early to mid-20^th^ century (Spears et al., 2022). For example, increases in eutrophication in lakes and rivers (Carpenter, 2005) led to bans on phosphate use in detergents in the U.S. and Canada by the 1980s (Litke, 1999), in a range of national and regional policy responses to limit phosphorus discharges from wastewater and agriculture (Spears et al., 2022), including laws on limited use of phosphates in Europe (European Commission, 2015). Increases in losses of phosphorus through agricultural practices and associated water pollution has focussed attention to phosphorus recovery. Recent technological developments, such as struvite crystallization from sewage to produce a recycled slow-release fertilizer (Le Corre et al., 2009) and enhanced biological phosphorus removal, show promise for phosphorus recovery from wastewater and agricultural runoff (Yetilmezsoy et al., 2017). While such advances make phosphorus recycling possible, upscaling challenges remain (e.g., cost-efficiency and availability of infrastructure), particularly in low Gross Domestic Product (GDP) countries. In addition to economic and logistical challenges, social barriers also impede the global application of phosphorus recycling and reuse (Withers et al., 2015). Policy interventions, cross-sector collaboration, and systems-level changes are therefore required.

Phosphorus interacts with nitrogen (N) and carbon (C) to drive greenhouse gas emissions leading to climate change, for example, driving methane emissions from polluted waterbodies (Beaulieu et al., 2019). At the same time, climate change affects the efficiency of fertilisers and the responses of ecosystems to pollution. These situations are highly dynamic and complex, they vary between countries and require local contexts to be clearly understood to ensure the effectiveness of sustainable phosphorus programmes.

Other key considerations must be taken including the level of potentially harmful impurities in both mined and recycled fertilisers. In this context, there is a need to closely monitor such impurities against intended product use. Of contemporary concern is the potential for cadmium accumulation in the soil associated with the use of fertilisers originating from some phosphate rock sources which may present a risk of toxicity in food systems (Six and Smolders, 2014).

### 1.2 The sustainable phosphorus summit series

This paper documents the unique and inclusive design of the 8^th^ Sustainable Phosphorus Summit (SPS8) in Africa, building on a series of 7 previous international SPS events. In addition, the paper highlights the collaborative and iterative process implemented for SPS8 Africa, a core focus of the Soil Advances Special Issue on Boosting Global Soil Science Collaboration. We then put forward the case for developing legacy outcomes, including an African Sustainable Phosphorus Network (ASPN). The ASPN will be a platform for stakeholders to collaborate with government departments and the private sector to develop policies that close phosphorus nutrient loops in Africa, thus ensuring sustainable food production and environmental protection.

**Fig. 1:**
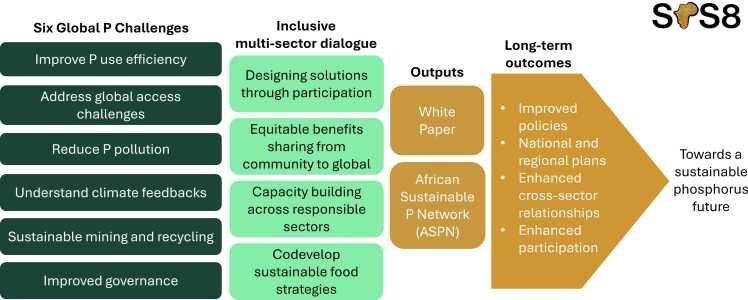
The 8^th^ Sustainable Phosphorus (SPS8) initiative Arrow. A conceptual diagram showing the component parts of the SPS8 initiative following a high-level Theory of Change Approach. The diagram works from the left where the SPS8 community is convened and the global challenges on sustainable phosphorus management co-developed. These global challenges are used to inform inclusive discourse within the SPS8 event with a focus on building sustainable solutions through partnerships in Africa. Key mid-term enabling outcomes from SPS8 are delivered to drive longer-term outcomes leading to behavioural change on sustainable phosphorus management from communities to global scales. P – phosphorus; SPS8 – 8^th^ Sustainable Phosphorus Summit, Accra, Ghana.

The SPS series is an established global platform focussed on phosphorus sustainability (Table 1). Since its inception in 2010, the SPS series has been held every 2–4 years on all continents – except Africa. The SPS is an international, multi-stakeholder conference to advance research, dialogue, networking, and action involving the sustainable use of phosphorus to improve food security, environmental integrity, and farmer livelihoods. Participants of previous SPSs have included both early career and senior researchers, entrepreneurs, policymakers, non-profit groups and other stakeholders who wished to gain and share phosphorus sustainability knowledge while lifting the voices of those who bring innovations to reality. In this paper we highlight the local context of sustainable phosphorus management in sub-Saharan Africa, outline the evolution of the SPS leading to SPS8 – Africa, and introduce co-development and capacity building initiatives being launched to enable the establishment of the first ASPN. Fig. 1 presents a summary of the global phosphorus challenges, the need for an inclusive multi-sector dialogue, which will be a core requisite of the ASPN, and expected long-term outcomes from these dialogues, required for realisation of the goal on sustainable management of phosphorus.

**Table 1:** Previous Sustainable Phosphorus Summits held between 2010 and 2022.

**Year**	**Summit Location**	**Main output(s)**	**Link**
2010	SPS1 Linkoping, Sweden	• Published Proceedings disseminated contents of papers presented	http://www.ep.liu.se/ecp/053/ecp10053.pdf
2011	SPS2 Tempe, USA	• Phosphorus, Food, and Our Future. Edited by K.A. Wyant, J.E. Corman, J.J. Elser. 2013. Oxford: Oxford University Press, 224 pp. • Developed important momentum to form the National Science Foundation (NSF) Research Coordination Network that provided a foundation for the new $25 M NSF Science and Technology Center on phosphorus sustainability (STEPS)	https://sustainability-innovation.asu.edu/events/rsvp/p-summit/
2012	SPS3 Sydney, Australia	• Blueprint for Global phosphorus security highlighting the principles, challenges and opportunities towards global phosphorus security and initiatives, strategies, roles and responsibilities of identified stakeholders.	SPS3_blueprint_for_globalpsecurity.pdf
2014	SPS4 Montpellier	• Special issue of Nutrient Cycling in Agroecosystems “Integrating Approaches to Sustainable Phosphorus Management in Agroecosystems” includes 11 articles.	http://sps2014.cirad.fr/
2016	SPS5 Kunming, China	• Forged collaborations and planning that led to production of “Our Phosphorus Future” report.	http://phosphorusfutures.net/announced−5th-sustainable-phosphorus-summit-to-take-place-in-kunming-china-august−2016/
2018	SPS6 Brasilia	• A published Proceedings disseminated abstracts of papers presented. • Development of priority areas for inclusion in the Our Phosphorus Future Report, bringing together the international scientific community to highlight priority actions on global sustainable phosphorus management. • Developed important momentum to form the Global Environment Facility uPcycle project, a $2 million project focussed on sustainable phosphorus management for lakes in Chile, the wider Latin America Region, and globally.	http://phosphorusfutures.net/announced−6th-sustainable-phosphorus-summit-brasilia-brazil-august−2018/ https://www.opfglobal.com/ https://www.upcyclelakes.org/
2022	SPS7 Raleigh-Durham, USA	• The Summit was part of the wider United States Phosphorus Week event run by the STEPs programme. Participation from over 200 stakeholders from the phosphorus value chain. • Discussions focused on challenges towards phosphorus sustainability, with a strong focus on Early Career Researcher development of the programme.	Presentations from this event available at: https://phosphorusalliance.org/get-involved/events/.

To date, hundreds of participants from around the world have joined seven SPS events (Table 1). Following SPS7 in 2022, the delegates stressed the obvious opportunity to hold SPS8 in Africa, where the need for food and farmer livelihood security is greatest. Previous SPS events have led to an increasingly connected international network of phosphorus experts and opportunities for professional development for graduate students and postdoctoral scholars. Specific event outcomes are presented in Table 1.

**Fig. 2:**
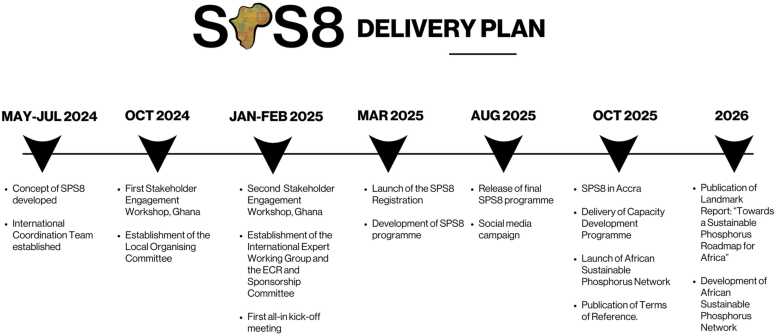
The 8^th^ Sustainable Phosphorus Summit (SPS8) Delivery Plan and Timeline. ECR - Early Career Researcher.

In the process of planning for SPS8 (Fig. 2), to be held Accra, Ghana, October 2025, the organising committee conducted over a 6-month period, a co-development process working with stakeholders (described in the working groups below) to identify 6 Priority Global Phosphorus Challenges. These Global Phosphorus Challenges were aligned with key questions or actions and helped to shape the structure of discussions during the summit.

The 6 SPS8 Global Phosphorus Challenges are:

1. **Phosphorus is critical in supporting global food security.** How can we improve the phosphorus use efficiency in food production systems?
2. **Access to phosphorus is varied across the world and we need to improve access and benefits where needed, e.g. Africa.** How can we address the imbalance in phosphorus fertiliser use?
3. **Phosphorus pollution of water can result in biodiversity loss and human health impacts.** How can we reduce the transfer of phosphorus from land to water and manage/mitigate its effects?
4. **There are complex interactions between phosphorus, nitrogen, carbon and climate.** We must increase our understanding and awareness of this, developing integrated mitigation and adaptation plans.
5. **Sustainable mining and recycling are essential to preserve phosphorus for future generations.** We must balance the supply of new phosphorus from mining, with increased attention to varied recycling streams.
6. **We need to improve our governance of phosphorus locally and globally.** We need to explore new opportunities for effective governances at multiple levels to help enact the transformations required.

## 2 The context of sustainable phosphorus management in sub-Saharan Africa

A key ambition of the SPS series is to highlight the specific contexts of the host region with regard to sustainable phosphorus challenges. While recognising that the Global Phosphorus Challenges above are shared, the SPS recognises that solutions to address them should be developed based on local knowledge and contexts. Below we provide a short review of evidence on the context in sub-Saharan Africa that was used to inform the development of SPS8.

### 2.1 Mineral fertiliser use in sub–Saharan Africa

Africa’s use of mineral fertiliser is sub-optimal, with average inorganic fertiliser application rates of 22.6 kg ha^-1^ on arable land (Elser and Haygarth, 2021, World Bank, 2021). This is irrespective of fertiliser type (i.e. N- or P-supplying fertiliser). Focussing on P, Sheldrick and Lingard (2004) reported a decline in phosphorus fertiliser use of about 233 %. This was based on historic data between 1961 and 1998. Later, Sattari et al. (2012) suggested that a five-fold increase in elemental phosphorus application rates is required by mid-century, with phosphorus fertiliser application rates increasing from 4 kg ha^-1^ in 2007 to 23 kg ha^-1^ by mid-century, if the region is to increase its food production. In addition to insufficient nutrient use/application, low nutrient use efficiencies reported on the continent are due to nutrient losses via leaching and volatilisation, especially in relation to N. For phosphorus, in addition to supply issues, the ability of most soils in Africa to tightly bind any added phosphorus is a widespread problem, meaning phosphorus fertilisers are often ineffective, especially on acidic soils. This leads to a “double burden” where Africa’s crop and food production potential is mainly limited by poor soils and a lack of financial capacity to access and apply optimal fertiliser rates, among other factors. Specifically, the soils have high levels of iron (Fe) and aluminium (Al) hydr(oxides) thereby binding phosphorus applied as fertilisers to these metals. Thus, the farmers are not deriving the maximum benefit in terms of yield per unit area of crops.

Fertiliser access in most developing countries is often constrained by the high prices relative to national incomes (i.e. using the GDP metric), albeit the existence of phosphate rock reserves in countries including Angola (Cabinda Phosphate), South Africa (Elandsfontein Phosphate) and Tanzania (Haneklaus et al., 2024), and a significant portion of the world’s phosphate reserves in Morrocco (OCP Group). For example, in Ghana, Kenya, Nigeria, and South Africa, the cost of fertilisers remains a significant challenge for smallholder farmers, accounting for 10–20 % of maize production costs in Kenya and 30–35 % in South Africa (IFPRI, 2022). In Ghana and Kenya, fertiliser costs almost doubled during the 2021–2022 crisis, increasing their share of production costs (IFPRI, 2022). This is against a relatively low GDP per capita (World Bank, 2021). In Nigeria, one of Africa’s largest oil producers, the lack of subsidies and reliance on imports often implies fertilisers are expensive, thus contributing to a large portion of agricultural costs (IFDC, 2020). Lastly, South Africa, while having a large and more diversified economy compared to many other African countries (World Bank, 2024a), still faces significant economic challenges (World Bank, 2024b) including making fertilisers affordable to rural farmers, with prices often outpacing income growth in many areas (FAO, 2021). This disparity highlights the difficulty smallholder farmers in these countries face in accessing the necessary inputs to boost productivity and food security.

Efforts in developing optimal fertiliser rates of phosphorus are often impeded by challenges in determining phosphorus fertiliser requirements for different soil/crop combinations. So, it is imperative to implement a widespread programme across Africa to determine optimal soil and crop specific phosphorus fertiliser application rates and alternative low-cost tests (i.e. dry spectroscopy).

### 2.2 Impacts of phosphorus pollution globally and in the context of sub-Saharan Africa

Over the past four decades, wastewater has been the dominant and growing source of phosphorus entering Africa's aquatic ecosystems (Malagó and Bouraoui, 2023). Reducing anthropogenic nutrient inputs from agricultural production and waste management into water bodies is a topical challenge. In 2015, the United Nations reported that about 500 coastal areas covering 250 000 km^2^ are eutrophic and hypoxic due to algal blooms from excess nutrients (UNDP, 2015). A recent study by Tigli et al. (2025) reported that rivers exceeding eutrophication thresholds (44 % in 2010) were projected to more than double by 2050 under a fossil-fuel development scenario which assumes globally high nutrient loads and high greenhouse gas emissions from human activities (Riahi et al., 2011, Strokal et al., 2021, Beusen et al., 2022). Specifically, using the Trophic State Index (TSI-Chla) by Carlson (1977), Tigli et al. (2025) reported a projected increase in algal blooms in 91 % of representative rivers (*n* > 3500) globally, by 2050, with the African region among continents projected to experience this change. In addition to effects of human activities on water resources, climate change has also been projected to have negative effects on water resources by 2050, thus damaging the ecosystem services acquired from these systems (Tigli et al., 2025). Indeed, climate change is already changing the behaviour of phosphorus as a pollutant in lakes across the world (Steinman and Spears, 2020). This is also likely the case in Africa where pollution from nutrients (and other sources including industry and plastics) may be exacerbated by climate change impacting on many ecosystem services (e.g. impacting human health, drinking water, aquaculture, tourism, fisheries etc; Poikane et al.,2024). It will be important to minimise the risks to freshwaters posed by the projected increases in human population and associated wastewater nutrient inputs coupled with the potential for increasing fertiliser application rates and soil-P loading across sub-Saharan Africa. This will require improvements in water resources monitoring, assessment, and reporting (following 1FAIR-ness principles) to inform restoration and protection efforts for high value ecosystems (Spears et al., 2021) whilst targeting innovation in phosphorus recovery and reuse options to protect essential water resources and their ecosystem services (Tammeorg et al., 2024).

### 2.3 Examples of sustainable soil management and phosphorus research in sub-Saharan Africa

Africa boasts some of the most locally adaptable soil fertility management options such as Integrated Soil Fertility Management (ISFM). ISFM has been widely promoted for over two decades to improve soil fertility and crop nutrition through use of organic nutrient resources (i.e. cattle/poultry manure, woodland leaf litter, N-fixing legume crops), improved seeds, balanced fertilisation, appropriate tillage technologies and local adaptations (Vanlauwe et al., 2015, Nezomba et al., 2015, Mugwe et al., 2019). Conservation Agriculture (CA) has also been widely promoted, mostly for improved water use efficiency and prevention of soil erosion and degradation caused by continuous ploughing. Both technologies have gained their importance in this region and are widely promoted by governments and Non-Governmental Agencies in selected countries including Zimbabwe under the *Pfumvudza* programme (FAO, 2023) and Malawi (Bell et al., 2018, Mwambungu, 2019, Manzeke-Kangara et al., 2025) through their agro-inputs subsidy programmes. However, the biggest challenge of degraded soils remains and some of the organic nutrient resources, especially cattle manure, has variable quality with some resources having very low phosphorus contents.

Studies conducted by different authors quantifying phosphorus contents of manure in different regions reported lowest phosphorus contents of 0.04 g kg^-1^ in West Africa (Harris, 2002) and similarly low phosphorus contents of between 0.059 and 0.063 g kg^-1^ in Southern Africa (Soropa et al., 2013). In contrast, relatively high phosphorus contents of between 0.20 and 1.61 g kg^-1^ in cattle manure was reported in East Africa (Lekasi et al., 2001). A study conducted by Zingore et al. (2008) reported that about 13.6–15.8 t of cattle manure ha^-1^ is required to supply recommended rates of phosphorus of 30 kg ha^-1^ in maize when cattle manure with relatively high phosphorus contents of 0.19–0.22 g kg^-1^ was applied. While repeated use of cattle manure contributes to fertiliser equivalents of ∼30 kg phosphorus ha^-1^ (Njoroge et al., 2019) and additional ancillary benefits of improved crop nutrition and dietary micronutrients (Manzeke et al., 2012, Manzeke-Kangara et al., 2021), use of lower quality cattle manure as reported by Harris (2002) would require much higher rates to be applied. This is not always practical for most smallholder farmers to achieve due to low numbers of livestock (Zingore et al., 2007). Additionally, most farmers only consider the amount of N when applying compound fertiliser. For example, Compound D contains variable formulations (i.e. 7 N:14 P_2_O_5_:7 K_2_O), and amounts applied are based on crop N requirements. Matching recommended rates of phosphorus by additional applications of straight phosphorus fertilisers such as Triple Superphosphate (TSP) and Single Superphosphate (SSP) is not commonly considered. This often leads to under-application of phosphorus, with N contributing 90 % of applied fertiliser (Sutton et al., 2013).

Phosphorus is an essential nutrient in plants. It regulates plant physiological responses to abiotic stress (i.e. heat, drought, water logging, high CO_2_ and salinity) (Lambers, 2022, Hawkesford et al., 2023, Khan et al., 2023) and plant biological processes i.e. reproduction and protein synthesis and energy supply for various cellular endergonic processes (Malhotra et al., 2018). Considerable research on phosphorus has been conducted in sub–Saharan Africa, in this context, with a major focus on grain legume crops due to their increased need for phosphorus in nodule formation (Kanonge et al., 2015, Hiama et al., 2019, Vanlauwe et al., 2019, Adjei-Nsiah and Ulzen, 2024).

The use of phosphate rock has been proposed as an option for improving soil fertility and crop productivity in acid soils (Sanchez et al., 1997) and is gaining momentum as a promising approach for improving soil fertility in Africa (IFDC, 2024) where the acidity of soils helps dissolve the phosphate. Over the past decade, research has also focused on the potential role of phosphorus applied using ISFM on grain nutrition and bioavailability of essential micronutrients such as zinc (Zn) (Manzeke et al., 2012). While the role of phosphorus in plant nutrition is widely recognised, its antagonism with micronutrients poses risks to plant Zn uptake and dietary Zn intakes in humans, especially in communities reliant on plant-based diets. This presents an imperative need for balanced phosphorus fertiliser application and conscious recognition of its potential inhibitory characteristics on other essential nutrients.

The phosphorus challenges are complex and urgent in sub-Saharan Africa and holding the 8^th^ SPS on the African content will put the spotlight on these issues and catalyse cross-sector actions to address them in Africa.

## 3 Supporting change through the sustainable phosphorus summit

### 3.1 Vision for the SPS8, Ghana, 2025

SPS8 aims to inspire and mobilise “Phosphorus Champions” to influence change across the region and build a network, culminating in an African Sustainable Phosphorus Network (ASPN). SPS8 will bring together the international and African phosphorus communities to deliver targeted capacity development activities during SPS8 in 2025. These activities will focus on enhancing pollution control while building resilience in food systems to climate change and enhancing sustainability benefits across the region. We will work with the ASPN to generate momentum and international profile, reputation and networking. The SPS8 will also be a platform to build capacity of early career researchers to lead discussions on sustainable phosphorus management and generate outputs (i.e. a White Paper covering various themes pertaining to phosphorus use and management). In this way, we will establish and diversify this network internationally targeting agricultural, environmental and social science academics, government officers, NGOs and UN bodies, in a partnership that delivers evidence-based change.

### 3.2 SPS8 inclusivity principles

The SPS will foster inclusive dialogue on major topics of concern for sustainable phosphorus management, set within the regional context of the hosts. The SPS acknowledges the need for science to support different approaches that are context specific, recognising cultural, scientific and policy differences and shared global challenges.

For the SPS8, the organising committees co-developed a set of Inclusivity Principles. It is our hope that these principles will help to guide the wider sustainable phosphorus community as they work together to address the major global challenges. The SPS8 Inclusivity Principles (in bold) and associated ambition statements include:

1. **Designing solutions based on inclusive participation across stakeholders.** The voices of all people and communities are fairly represented in the design of solutions to deliver sustainable phosphorus management, including those from marginalized groups. SPS8 is fulfilling this by ensuring inclusive representation at the Summit, offering support for participants from low-income countries and early-career researchers.
2. **Ensuring benefits are shared equitably from communities to global scales.** Sustainable phosphorus management ensures that its benefits reach vulnerable communities while extending positive impacts beyond the immediate scale of intervention. The outputs of SPS8 will be widely shared, spanning local to global communities, including identifying opportunities to maximise benefits for vulnerable stakeholders including smallholder farmers. The form of these materials will be collaboratively tailored to meet accessibility and literacy standards.
3. **Building capacity across responsible industries and institutions**. Capacity development is focused on industries, institutions and regulatory bodies who have a role in implementing sustainable phosphorus management measures. SPS8 is fulfilling this by fostering transparent, equitable and respectful dialogue across sectors to prioritise training and knowledge exchange needs.
4. **Contributing to a global strategy to ensure sustainable food production in sub-Saharan Africa.** Sustainable phosphorus management is a global responsibility essential for advancing sustainable food production and resilience while minimizing losses and damages associated with environmental degradation, both nationally and internationally. The SPS8 is championing this effort by uniting local and international participants, along with Expert Working Groups, to embrace a collective responsibility for producing food sustainably while minimising environmental harm.

### 3.3 Building the SPS8 network

The SPS8 has convened committees and special working groups to set the scientific agenda so that major outputs can be supported, securing a long-term impact and legacy on the global scale. These groups are designed to foster knowledge exchange around the delivery of the SPS8 vision. A series of engagement activities were planned and undertaken by the convening institutions of the International Coordination Team in a two-year programme commencing from 2024 to 2026 (Fig. 2). This programme was designed to build momentum and collaboration across African and international communities to develop the ‘Delivery Plan for the SPS8 Vision’ and to ensure a lasting legacy.

The following groups have been established:

**The International Coordination Team (ICT)**. The ICT is Co-Chaired by representatives of the primary convening Institutes including Lancaster University, the UK Centre for Ecology and Hydrology (including the UKCEH West Africa Office, Accra, Ghana), and Rothamsted Research, with representation from all subsequent Committees. Some members of the ICT (including but not limited to M.G.M.-K. and A.A.A.) have extensive research experience of working with different agricultural stakeholders including smallholder farmers and agricultural extension staff. The ICT takes responsibility for convening all committees, for managing knowledge exchange and collaboration between them, and for managing budgets and SPS8 logistics including the summit venue, registration, communication and onboarding sponsors. The ICT is responsible for fostering diversity and inclusivity in the delivery of the SPS8.

**The Local Organising Committee (LOC)**. The LOC is Chaired by representatives from the Soil Research Institute, Kumasi, Ghana and includes representation from all major research and policy institutions in Ghana, including universities, government research centres, and relevant government ministries. The LOC is responsible for setting the technical agenda of SPS8 ensuring that local contexts are appropriately reflected, and for engaging with regional scientific, NGO, industry, and policy communities in the West Africa Region and beyond. The LOC is responsible for setting the agenda for targeted discussion sessions associated with the SPS8 and for shaping the legacy outcomes, including the African Sustainable Phosphorus Network.

**The International Expert Working Group (IEWG)**. The IEWG is Co-Chaired by representatives of Arizona State University, USA, the University of Technology Sydney, Australia, and Rothamsted Research, UK. Some members of the IEWG (i.e. Z.O.-U.) have extensive research experience in sub-Saharan Africa and thus provide knowledge on context specific phosphorus challenges. The IEWG convenes global experts in the field of sustainable phosphorus management across Asia, Latin America, Europe, North America and Australia-New Zealand. It is responsible for co-developing the technical programme, chairing the technical sessions, and peer-reviewing abstracts and selecting speakers, a responsibility they share with the LOC. Members of the IEWG work collectively to raise awareness of the SPS8 initiative, and to identify opportunities for financial support for participants.

**The Early Career Researcher and Sponsorship Working Group (ECRG).** The ECRG is Co-Chaired by representatives of the Mohammed VI Polytechnic University, Morocco, University of Cape Coast, Ghana, and North Carolina State University, USA. The ECRG are responsible for supporting opportunities for participation in the SPS8 by early career researchers to attend the conference, ensuring representations from across Africa and internationally. The co-chairs work together to set the ECR-agenda in the programme and to match participants with sponsorship opportunities.

### 3.4 Supporting a lasting legacy following SPS8

**Fig. 3:**
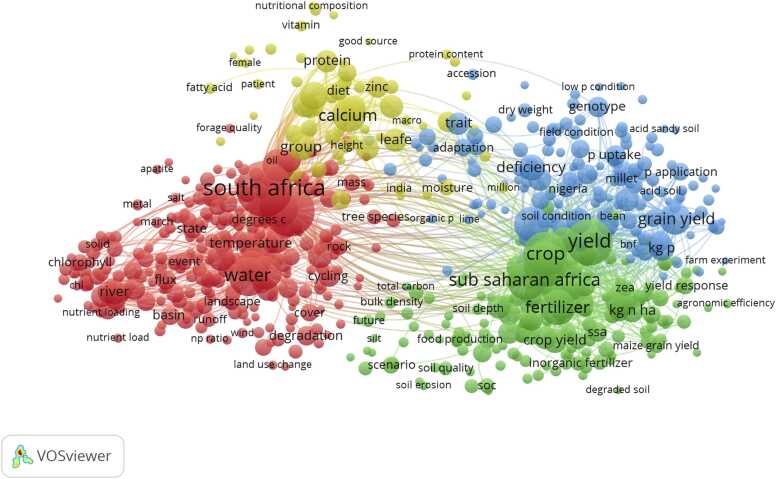
Term map of the most relevant terms the in titles and abstracts from the search input ("Phosphorus") AND ("Africa" or "sub–Saharan Africa" or "sub-Saharan Africa" or "Sub Saharan Africa" or "Sub-Saharan Africa"). Search conducted on 30 January 2025. Created using VOSviewer version 1.6.20 downloadable at https://www.vosviewer.com/. Key on Topic grouping: Red: Environmental focus; Green: Soil fertility and Crop productivity; Blue: Soil status and Conditioning; Yellow: Human nutrition. bnf-biological nitrogen fixation, zea-Genus name for maize. March-the month of the year, np ratio-Nitrogen to Phosphorus ratio.

**Fig. 4:**
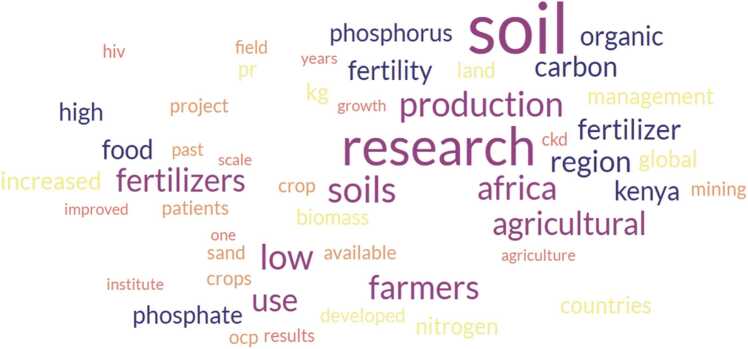
Top 50 words from 12 manuscript generated using the search terms (“Phosphorus” AND “* Africa”) AND (“Collaborat*”) in Web of Science. pr-phosphate rock, ckd-chronic kidney disease, one-the number (1), OCP- the world's largest phosphate mining and processing company based in Morocco. WordCloud generated using https://www.freewordcloudgenerator.com/.

A key focus of SPS8 is to support the co-development of legacy outcomes, so that the African community is enabled to meet the global challenges outlined above. A clear focus has been on connecting the African community with the international community in this field. The ICT visited research institutes in Ghana working on soil and crop research and water management in 2024 and 2025 to gather contextual evidence of current research topics and priorities with which to inform the international community. This is supplemented, here, with an analysis of contemporary research activity on sustainable phosphorus research focussed on Africa, but not exclusively conducted by researchers based in Africa (Box 1; Fig. 3, Fig. 4). A brief description of key research topics and issues identified during these visits related to phosphorus management in soils, crops and water systems is presented in Table 2.

Box 1 **Analysis of the Scientific Literature on Sustainable Phosphorus Research in Africa.**

A Web of Science search was conducted to ascertain the number of publications working on phosphorus in Africa, using a collaborative approach towards sustainable and inclusive research. The search terms were (“Phosphorus” OR “Africa” OR “Sustaina*” OR “Inclusiv*”) AND (“Collaborat*”). The term Sustaina* was used to include studies using the words sustainability, sustainable in the abstracts. Similarly, the term Inclusiv* was used to include studies using the words inclusivity, or inclusive in the abstract and Collaborat* to include abstracts with collaboration, collaborate, collaborating etc. This input search had an output of 48 576 abstracts as of 20 November 2024. We discarded this search because using the search terms (“Phosphorus” OR “Africa” OR “Sustaina*” OR “Inclusiv*”) AND (“Collaborat*”) would produce studies working on different studies for example sustainability and collaboration without a specific focus on P. We then conducted a more specific search with input terms ("Phosphorus") AND ("Africa" or "sub–Saharan Africa" or "sub-Saharan Africa" or "Sub Saharan Africa" or "Sub-Saharan Africa"). This had an output of 2 012 abstracts (as of 30 January 2025), published between 1973 and 2025. Most of the abstracts (*n* = 414) were in the Agronomy research area followed by Environmental Sciences (*n* = 359), Soil Science (*n* = 356), Plant Sciences (*n* = 353) and Ecology (*n* = 253). Agriculture multidisciplinary, Water Resources, Geosciences multidisciplinary, Multidisciplinary Sciences and Marine Fresh Water Biology had comparable abstract numbers of 149, 135, 111, 103 and 101, respectively. The search output also showed recent increases in phosphorus research as most abstracts (*n* = 811) were published between 2018 and 2024, with > 100 abstracts published per year. Fig. 3 shows a term map of the most relevant terms in the titles and abstracts of the 2 012 articles. Each circle represents a term from the titles and abstracts of publications in this dataset. The terms are located based on the co-occurrences in the titles and abstracts. The higher the co-occurrence of two terms, the closer they will be in the map for example grain yield and kg P.

While this was a promising output on phosphorus research in Africa, not many studies focused on collaborative efforts for sustainable management. This was evidenced by a rather low output of *n* = 12 abstracts when the search terms (“Phosphorus” AND “Africa”) AND (“Collaborat*”) were used on the 30^th^ of January 2025. Fig. 4 shows the top 50 words from the 12 abstracts. Interestingly much focus was on soil research. An analysis of abstracts by sector in Web of Science confirmed this, reporting the number of abstracts per sector as follows: Agriculture (including Agronomy and Soil Science) (n = 8), Science Technology and other topics (n = 2), with Business Economics, Geology, Operations Research Management Science and Urology Nephrology contributing one abstract each. This shows that while research on phosphorus is widely conducted, many challenges remain including the need for collaborative research efforts and capacity building within the region and beyond. Capacity building has recently been identified as one of the main components required for increasing the self-reliance and sustainability of research in Africa (Manzeke-Kangara et al., 2024). However, due to limited financial capacity of local Governments to fund doctoral training programs, research capacity building in Africa, ongoing investment and collaboration with international partners could contribute towards sustainable research in Africa and consequently food self-sufficiency. With projected increases in population of 2.7 billion people in Africa alone (Canning et al., 2015) which would represent approximately 58 % of the global population growth (Worldometers, 2019; Elrys et al., 2020), self-sufficient and sustainable research in Africa is of paramount importance.

**Table 2:** Key phosphorus related issues compiled during consultations in 2024 and 2025 with academic and research leaders in Ghana, Africa.

**Organisation**	**Subject/Topics**	**Key issues identified related to phosphorus**
The Soil Research Institute, CSIR	• Provide agricultural support and advice to farmers. • Conduct on-farm field demonstration trials and mobile soil testing.	• Phosphorus deficiency in soils. • Highly acidic soils. Poor crop productivity. • Fruit abortion in horticultural crops. Lack of awareness about phosphorus limitation in Ghanaian soils Lack of resources for *in situ* data collection.
The Water Research Institute, CSIR	• Conducts water resources research. • Provide scientific and technical information and services related to water and related resources. • Provide strategies for the sustainable development, utilization and management of water resources for the socio-economic advancement of Ghana.	• Decade-long monitoring of Lake Volta, which is ongoing and occurs twice a year. • Lake Volta, the world’s largest artificial reservoir by surface area, is projected to be prone to eutrophication due to stream bank cultivation.
The Aquaculture Research and Development Centre (ARDEC), an outstation in Akosombo under the CSIR-WRI	• Conducts research on biotic and abiotic factors affecting aquaculture (fin fish and oysters). • conducts diverse research on tilapia (Oreochromis) breeding, growth, and disease resistance (Magna et al., 2024). • Supports fish farming farmers in Ghana.	• ARDEC implements sustainable fish farming and water management practices (i.e. use of bacteria to build fish immunity in place of antibiotics, use of lettuce for wastewater recycling and reuse, use of plant-based fish additives (i.e. ginger and garlic) as feed and to control infection in fish (i.e. dried papaya leaves). • ARDEC recycles phosphorus waste from fish farming into nearby banana plantations. • Potential phosphorus pollution due to encroachment of human activities (i.e. agriculture and sewage management) into the Volta River. • Research to explore cyanobacterial blooms’ threshold required.
The Environment and Sustainable Development Department, Volta River Authority	• Aquatic waste management, biannual greenhouse gas inventories, water quality monitoring, and salinity monitoring.	• Pollution “hotspots” around the reservoir, including a textile industry and slaughterhouse. • Opportunities exist to expand monitoring “in” sources of phosphorus for example the amount of fertiliser inputs into the Volta River. • Sustainable phosphorus management strategies including harvesting and composting of water hyacinth and other weeds from the river (Volta River Authority, 2023). • Growing water hyacinth in eutrophied fishponds to recover nutrients. • Use of compost as pellets, in banana and rice plantations. • Phosphorus monitoring in harvested weeds and compost to estimate phosphorus generated from organic waste.

CSIR: Council for Scientific and Industrial Research; CSIR-WRI: Council for Scientific and Industrial Research-Water Research Institute.

A focus of the discussion sessions in SPS8 will be to develop a coherent long-term Roadmap for Sustainable Phosphorus Management in Africa (i.e., in the form of a White Paper), drawing on perspectives from all stakeholders. The following priority outcomes were identified during the Stakeholder Engagement Workshops. These priority outcomes will be developed further during the SPS8 and through the production of the White Paper.

Key legacy outcomes and actions proposed to progress them.

− Enabling a new generation of African Sustainable Phosphorus Experts through implementation of African led capacity building Doctoral training programmes and exchange visits.
− Establishing an African Sustainable Phosphorus Network. The Network will be launched as part of the 8^th^ SPS agenda in Accra, Ghana, to enable collaborations and networking among stakeholders working in the agriculture, industry and water sectors. The African Sustainable Phosphorus Network will also present an opportunity to identify and deliver capacity building programmes.
− Delivering a high-level Roadmap for Sustainable Phosphorus Management in Africa. This will be implemented through a series of workshops to map what needs to happen for phosphorus fertilisers to be available for use in arable cropping and soils (i.e. phosphorus bioavailability), to identify options for reducing losses from wastewater and agriculture to the water environment, and to inform ecosystem recovery responses to address pollution impacts. Attention will also be given towards identifying opportunities for phosphorus recycling in Africa and ensuring ongoing legacy discussions to advocate for the African Sustainable Phosphorus Network to be recognized within the African Soil Strategy through consultation with relevant Development Bodies (e.g., the African Union).
− Clarifying the policy development opportunities and priorities to improve sustainable phosphorus management across Africa.

## 4 Conclusion

The projected global population growth and the need to transform sustainable food production to achieve food security is envisioned to pose extreme environmental challenges; globally and across Africa. Extensive research on phosphorus use in arable cropping has been widely implemented in Africa, albeit with room for improvement on collaborative research on phosphorus sustainability and on environmental impacts. The scientific evidence on sustainable phosphorus management in Africa, and sub-Saharan Africa in particular, highlights some regional contexts (e.g. on soil biogeochemistry and phosphorus bioavailability and on fertiliser application and accessibility constraints) that must be considered when developing a Road Map for Sustainable Phosphorus Management in the region. This includes projections of worsening phosphorus pollution of freshwater systems. While examples of sustainable phosphorus measures in Africa exist, including monitoring of water resources for eutrophication and recycling of biomass waste for use in arable cropping, these are often implemented at pilot scale. Nevertheless, increasing fertiliser accessibility and optimising application remains a key priority to ensure improvements in farmer livelihoods and food security in the region. There is, therefore, a need to scale-up sustainable phosphorus use initiatives within the region through collaboration, networking and training opportunities to generate robust scientific evidence to underpin effective supporting policies. The SPS8 initiative is working collaboratively to identify and address the priority issues and actions needed to enhance sustainable phosphorus management in the region. The development of an African Sustainable Phosphorus Network should stand to support this ambition into the future.

## CRediT authorship contribution statement

**M.G. Manzeke-Kangara:** Validation, Writing – original draft, Conceptualization, Writing – review & editing, Data curation, Visualization. **F.M. Tetteh:** Conceptualization, Writing – review & editing. **E.K. Gbekor:** Writing – review & editing. **J.J. Elser:** Writing – review & editing. **D. Cordell:** Writing – review & editing, Conceptualization. **A.A. Asante:** Investigation, Writing – review & editing, Conceptualization, Project administration. **K.A. Frimpong:** Writing – review & editing. **E. Zaja:** Writing – original draft, Project administration, Visualization, Writing – review & editing, Conceptualization. **W.J. Brownlie:** Writing – review & editing, Project administration, Writing – original draft. **M.S.A. Blackwell:** Writing – original draft, Writing – review & editing. **M. Owusu:** Conceptualization, Writing – review & editing. **E.K. Nartey:** Writing – review & editing. **M. Miyittah:** Writing – review & editing. **V. Logah:** Writing – review & editing. **I.H. Lewis:** Writing – review & editing, Project administration. **J.X. Kugbe:** Writing – review & editing. **B.M. Spears:** Writing – review & editing, Conceptualization, Validation, Writing – original draft, Software. **D. Touhami:** Conceptualization, Writing – review & editing. **P.M. Haygarth:** Supervision, Conceptualization, Writing – original draft, Project administration, Writing – review & editing, Resources, Validation, Funding acquisition. **K. Harris-Gilliam:** Writing – review & editing. **C.K. Tay:** Project administration, Conceptualization, Writing – review & editing. **Z. Oyetunde-Usman:** Writing – review & editing.

## Author contributions

M.G.M.-K. and P.M.H. proposed the manuscript for submission to the Special Issue. M.G.M.-K., E.Z. and B.M.S. led the preparation of the paper outline, with initial draft development contributions from M.S.A.B. and W.J.B. Data analyses and visualizations presented in this manuscript were prepared by M.G.M.-K., B.M.S., and E.Z. P.M.H. led the development of the Global Phosphorus Challenges. [A.A.A]. coordinated the consultation of phosphorus researcher priorities in Ghana. All authors contributed to further iterations of the manuscript towards the final version submitted.

## Funding

The author(s) declare financial support was received for organisation of the 8^th^ Sustainable Phosphorus Summit from 10.13039/100010029Lancaster University and UKCEH, with additional funding to be sourced from delegate registration and sponsorship. Lancaster University supports the project through Global Advancement Funding (project 053, OVE 1038). UKCEH researchers were supported by the GEF-UNEP uPcycle Project and through an Agile Grant associated with the 10.13039/100014013UK Research and Innovation (UKRI) 10.13039/501100000270Natural Environment Research Council (NERC) through their National Capability for International Science Programme (UKCEH International Science for Net Zero+). Rothamsted Research receives strategic funding from 10.13039/501100000268BBSRC; Rothamsted contributors acknowledge support from the Growing Health (BB/X010953/1) and the Resilient Farming Futures (BB/X010961/1) Institute Strategic Programmes. J.J. Elser's contribution was supported by the Science and Technologies for Phosphorus Sustainability (STEPS) Center under 10.13039/100000001National Science Foundation (NSF) award number CBET-2019435. Some authors receive funding from industry to support their research while others represent industry bodies. However, these authors declare no competing interest and the content of the paper does not necessarily reflect the position of any individual affiliated body or institution.

## Declaration of Competing Interest

The authors declare that they have no known competing financial interests or personal relationships that could have appeared to influence the work reported in this paper.

## Data Availability

No data was used for the research described in the article.

## Footnotes

1 Findable, Accessible, Interoperable, Reusable
